# Our Sympathy

**Published:** 1891-03

**Authors:** 


### OUR SYMPATHY.

---

Our valued New York correspondent, Dr. William L. Russell, writes that he was unable to furnish his usual letter, owing to the death of his little boy.

The JOURNAL extends its most sincere sympathy to the Doctor and his wife in their great sorrow, and hopes Time will kindly heal the wound of so severe a loss until they may become somewhat resigned to their affliction.

### ITEMS.

LATE DEVELOPMENT OF HYDROPHOBIA.—W. G. Spencer, M. S., reports (*British Medical Journal*, Feb. 7, 1891) the longest authentic period of time elapsing between the infliction of a bite by a rabid animal and the development of hydrophobia, viz.: two years and four months.

---

Eight patients are being treated in the Post-Graduate Hospital by Koch's lymph. Three of them are cases of lupus; four are cases of phthisis pulmonalis, and one laryngeal tuberculosis. The inoculations are in charge of Dr. W. C. Bailey, who was for a long time a student in Koch's laboratory, assisted by the director of the laboratory, Dr. J. H. Linsley.

---

We learn from the *Medical Record* that a great deal of medical legislation is being done out in California just now.

It is proposed to appoint a State Veterinary Surgeon. Also to regulate the practice of pharmacy and the sale of poisons; to establish a State board of funeral directors; to regulate the practice of veterinary medicines; to forbid the sale of cigarettes to boys; to appropriate $80,000 to the medical department of the University of San Francisco. All of which is probably well enough; but we modestly suggest to the regulating law makers the appointment of a Board of Medical Examiners. As a well "regulated" State, California would then be a distinguished success.

---

In a late *Medical Record* (Feb. 21) Dr. A. M. Phelps, of New York, describes in detail his recent attempt at grafting the bone of a dog to the tibia of a boy, for the treatment of ununited fracture.

The operation consisted in freshening the ends of the bones and inserting between them about an inch of the dog's radius, the nutrient artery supplying the latter being preserved.

This operation has been considerably discussed in the secular press, and, as usual, there was not lacking some over-humane individuals who looked upon the procedure only as a cruel experiment upon a dog.

So far as affording relief to the patient is concerned, the operation was not a success. Sufficient time (only eleven days) was not given for bony union to occur ; but nature was evidently doing her best. On the whole, the result was encouraging, and we hope subsequent trials will be more successful.

---

THE MISSISSIPPI VALLEY MEDICAL ASSOCIATION will hold its seventeenth annual session at St. Louis, Wednesday, Thursday and Friday, October 14, 15 and 16, 1891. A large attendance, a valuable programme and a good time are expected, The members of the medical profession are respectfully invited to attend.

C. H. HUGHES, M. D., President,
500 N. Jefferson Ave., St. Louis.
E. S. MCKEE, M. D., Secretary,
57 West Seventh St., Cincinnati.
I. N. LOVE, M. D., Chair. Com. of Ar.,
301 N. Grand Avenue, St. Louis.

---

### GOLD AND MANGANESE IN TUBERCULOSIS.

On Tuesday evening last, before the Section in General Medicine of the New York Academy of Medicine, Dr. J. B. White, physician to Charity Hospital, read a paper on the therapeutic value of gold and manganese when subcutaneously administered to patients suffering from pulmonary or other forms of tuberculosis. He makes use of a solution each drop of which was said to represent one fiftieth of a grain of some salt or salts of the metals. For injection one or two drops of this are added to five or ten minims of a one per cent. solution of carbolic acid. The administration of the remedy is followed by a pronounced reaction in its general characteristics similar to that produced by Koch's liquid. Dr. White narrated the histories of a number of pulmonary cases treated by him at Charity Hospital, from which it might be deduced that the expectoration has been remarkably lessened in quantity, in one instance from fourteen ounces to two in twenty-four hours. An increase of appetite had been observed in all the cases. The improvement in respiration had not been so immediately apparent as the other evidences of general amelioration, but the patients had, nevertheless, all been more or less relieved in their breathing. One of the most encouraging results had been the decided effect upon the functions of nutrition, as shown by the increase of weight. In one instance this had been as much as seven or eight pounds in a few weeks. Without exception, the patients had expressed themselves as feeling better under the treatment. The speaker, who was corroborated by several colleagues who had followed his experiments, said that he did not wish to be understood as urging any specific virtue for this remedy, but thought that further trial was likely to demonstrate its value as an adjuvant to the general treatment of tuberculous cases. —*The New York Medical Journal, February* 21, 1891.

---

### REMOVAL OF A PORTION OF THE LIVER WITH THE ELASTIC LIGATURE.

At a meeting of the Académie de Médecine of Paris on January 7th, M. Simon Duplay reported on a case in which M. Terrillon had removed by means of an elastic tube a portion of the liver containing numerous hydatid cysts of small size. The patient (a woman, aged 53) had for six years suffered from frequently repeated attacks of sharp pain in the right side, and had noticed for some time a slowly increasing tumor in the region of the liver. On August 1st, 1889, laparotomy was performed by an incision about six inches in length parallel to the lower border of the false ribs on the right side. On subsequent enlargement of this wound and free exposure of the liver, a portion of the right lobe, about the size of two fists put together, was found to be studded with numerous small cysts. There was a very sharp line of demarcation between the healthy and diseased portions of the hepatic tissue. After an unsuccessful atttempt to remove the diseased portion of liver by the thermo-cautery, the use of which was followed by much bleeding, M. Terrillon constricted the base of the growth by a rubber tube. The strangulated portion of the liver, which was fixed outside the wound in the abdominal wall, gradually mortified, and, after the removal of the ligature on the seventh day, was cut away. The large open surface that was left soon healed, and ultimately the patient made a complete recovery. In his remarks on this case, M. Duplay stated that several instances had been recorded of removal of a more or less considerable portion of the liver, such treatment having been practised in accordance with one or the other of two very different indications. The surgeon has had in some cases to deal with a hernia of this organ through a penetrating wound of the abdominal wall, and to remove by the knife the protruded portion; and in other cases removal has been performed after laparotomy, with the object of extirpating a hepatic tumor. Of the former class of cases, about ten instances have been recorded, in the majority of which, notwithstanding an absence of antiseptic precautions, the patients recovered. Very few cases, however, probably not more than three, have been published in which removal of a new growth had been attempted. In each of these the operation was attended by alarming and, indeed, in one instance, fatal hemorrhage. As an ordinary and non-elastic ligature usually tears through the friable hepatic tissue, the application of an elastic ligature, as suggested and practised by M. Terrillon, constitutes, M. Duplay holds, a decided progress in the *technique* of resection of the liver. This method of hæmostasis is applicable not only to pedunculated but also to sessile tumors, in which later the elastic ligature fulfils a double object: one of producing, through constriction of the tissue of the liver, a kind of artificial pedicle, which permits exact circumscription of the diseased part, and facilitates its subsequent removal by the knife; the other of very effectually securing the patient against the risks of hemorrhage.—*Supplement to the British Medical Journal, February* 7, 1891.

### ADULTERATION OF DRUGS.

---

We call attention to the following circular letter:

GEORGIA STATE BOARD PHARMACY,<br>

OFFICE OF SECRETARY,<br>

LAGRANGE, GA., March, 1891.

H. R. SLACK, Analytical Chemist. MEMBERS: John W. Goodwyn, Chairman, Macon; H. R. Slack, Secretary, LaGrange; S. C. Durban, Augusta; Harry Sharp, Atlanta; F. Joerger, Brunswick.

The Georgia State Board of Pharmacy has issued the following letter to the druggists:

DEAR SIR—Your attention is respectfully called to the law against adulteration, which was passed by the Legislature, in 1889, but has not been enforced because of lack of funds.

### ADULTERATION LAW.

SEC. 9. No person shall within this State manufacture for sale, offer for sale, or sell any drug, medicine, chemical or pharmaceutical preparation which is adulterated. A drug, medicine, chemical or pharmaceutical preparation shall be deemed to be adulterated: (1) If, when sold under or by a name recognized in the U. S. Pharmacopœia, it differs from the standard in strength, quality or purity laid down therein. (2) If, when sold under or by a name not recognized in the U. S. Pharmacopœia, but which is found in some other standard work, it differs materially from the standard of strength, quality or purity laid down in such work. (3) If its strength, quality or purity falls belowed the professed standard.

The last Legislature made the necessary appropriation to enable the Board to execute the law.

The Board intends to buy up samples of drugs from different sections of the State and have them analyzed. If analyses show goods to be adulterated, the parties selling them will be prosecuted.

The fine is $100 and the expense of analysis to be added.

The law makes you responsible, not only for the goods you make yourself, but also all that you sell manufactured by others; you therefore see the necessity of making tinctures, etc., from full strength goods, and buying drugs and chemicals of strictly reliable firms.

Wholesale druggists are especially warned against making Grocerymen's Laudanum. You can make half-strength goods, but you must so label them or suffer the consequences.

Hoping you will give this your attention and help us in our efforts to elevate the standard of pharmacy in our State, we are,

Yours truly,

JOHN W. GOODWYN, Chairman.

H. R. SLACK, Secretary.

P. S.—The next meeting of the Board will be in Augusta, May 11th. The Georgia Pharmaceutical Association meets there the next day. Hope you will attend.

---

### CONSTITUTION—THE UNITED STATES MEDICAL PRACTITIONERS' PROTECTIVE ALLIANCE.

FOUNDER: Dr. J. H. DeWolf, Baltimore, Md.; President, Dr. W. H. Crim, Baltimore, Md.; Vice-President, Dr. W. V. Wilson, West Haven, Conn.; Secretary, Dr. J. F. Davidson, Glendola, N. J.; Treasurer, Dr. R. B. Elderdice, McKnightstown, Pa.

### ARTICLE I.

This Society shall be known as the United States Medical Practitioners' Protective Alliance.

### ARTICLE II.

The object of this Association shall be to maintain organized co-operation amongst the practicing physicians, who are legally qualified to practice in their respective States, and in good standing in the profession; for the purpose of protecting medical practitioners from the abuse of dispensaries that treat many who are well able to pay; from the unjust competition caused by short term, quick graduating, and inferior Medical Colleges. To endeavor to promote the passage of just and equitable laws, regulating the practice of medicine in all the States, so that the license to practice issued in any one State shall be valid in any other State; and to devise means to enhance our financial condition (and thereby our usefulness) in every honorable way, and to derive the incalculable benefits that only can be obtained by combination and unity of action.

ARTICLE III.

The members of this Association shall exercise toward each other, toward all physicians and toward all mankind that courtesy and just dealing to which every one in his legitimate sphere is entitled, and any departure therefrom shall be deemed unprofessional, undignified and unworthy the honorable practitioner. It shall also be regarded as unbecoming to engage in any form of advertising or practice which shall tend to lower the physician in the esteem of the community or reflect discredit upon his professional associates.

ARTICLE IV.

The officers of this Association shall consist of a President, Vice-President, Treasurer and Secretary, who shall be elected annually. The seal of this Association must be stamped on all official papers.

ARTICLE V.

The fees for membership shall be three ($3) dollars on admission, and two ($2) dollars per annum thereafter. Delinquent members to be dropped for non-payment of dues whenever in arrears over two years.

ARTICLE VI.

Any member may be officially censured, invited to withdraw, or be expelled from membership for improper conduct or violation of professional comity. But it shall be necessary for a specific charge to be made in writing, with the name of accuser, and a copy to be presented to the accused, or some person acting in his behalf, and another placed in the hands of the President or Secretary one month before the time of holding a regular meeting, when ample opportunity will be given for a trial.

ARTICLE VII.

That direct appeals be made by us as a body to the Legislatures of our various States, from time to time, as may be deemed expedient, to secure the repeal of unjust or obnoxious laws which may be in existence, or the passage of laws that are vital to our success and our welfare as a profession.

ARTICLE VIII.

That an effort be made to secure a law in each State that will secure the medical practitioner from the many losses he now sustains from those able to pay, but unwilling, and to make the physician's claim in all cases a preferred one, which must be paid before the *pro rata* of an estate, as is now the law in some of the most enlightened countries of Europe.

Those desirous of joining address Dr. J. H. DeWolf, 1600 Franklin street, Baltimore, Md. Please enclose stamp.

---

PITRES (M.) "De la neurasthenie et l'hystero-neurasthenie traumatique."—*Le Progrès Medical*, December 6th, 1890.

A SEVERE shaking may determine various nervous affections. One patient may present symptoms of true hysteria ; another those of ordinary paralysis agitans; a third may suffer from neurasthenia ; while a fourth becomes epileptic.

M. Pitres then reviews the symptoms of ordinary spontaneous neurasthenia. He classifies it under the following forms : (1) Cerebral, in which headache (worse by day) is the chief symptom; (2) spinal, with pain in the back, and peculiar sensations in the legs; (3) neuralgic; (4) cardiac, with palpitation and attacks of angina; (5) gastro-intestinal, with dyspepsia and constipation; (6) a group of cases in which the symptoms are referable to the reproductive organs. These divisions are, of course, more or less artifical, for the symptoms rarely belong exclusively to one or other of them. The mental state is nearly always the same—the patient is restless, irritable, and emotional. He reasons about his condition, believes himself suffering from organic disease, and finds pleasure in talking about his ailment. This disease is more common in men between the ages of 20 and 50, and in those of neuropathic tendencies.

Neurasthenia may follow railway accidents. M. Vibert has found in most cases a state of nervous excitement after these accidents, characterized by sleeplessness, headache, tremblings and excitability. These systems may quickly disappear, but they may last a long time.

The prognosis of traumatic neurasthenia is difficult to give. Recovery is never rapid. In the more favorable cases the disease may last from two to three months, in others, years.

M. Pitres then relates the following cases:—

(1) *Neurasthenia of traumatic origin.*—Man, æt. 25. Fall through fifteen feet. When he regained consciousness, he complained of headache, and vomited. In three weeks' time he got up, and left the hospital shortly after. A little later he was taken with restlessness, and sadness, and headache. Two months after the accident he had constant headache, his gait was unsteady, and although used to scaffolding, he felt uneasy when standing on a chair. He was irritable and morose. This patient recovered.

(2) *Hystero-neurasthenia of traumatic origin.*—Man, æt. 34. Fall through sixty feet. There was some difficulty in articulation shortly after, and sensation on the palate and tongue was diminished. This persisted, and three weeks later there was added tremor of the face muscles, a total right sided hemi-analgesia, and a concentric diminution of the field of vision on both sides. He was sleepless and very irritable. The hemi-analgesia disappeared suddenly, and after hydropathic treatment the cure was complete. The hemi-analgesia, difficulty of articulation, loss of pharyngeal reflex, and concentric diminution of the field of vision were due to hysteria; the mental change, headache, and insomnia, to neurasthenia.

(3) *Symptoms of hysteria, neurasthenia, and Graves's disease.*—Man, æt. 35. Fall through twenty-four feet. Three months afterwards he began to feel fatigue at his work, he could not fix his attention, and walked with difficulty in the dark. He also suffered from palpitation, and his eyeballs became too prominent. He had the globus hystericus. Three months later he had to give up his work. He was depressed, with suicidal tendencies, and suffered from headache. Fourteen months after the accident he was still melancholic, and the headache persisted. His skin and mucous membranes were anæsthetic. Eyeballs prominent, monocular diplopia, both fields of vision concentrically limited. Rapid vibratory tremor in the head and all four extremities. Thyroid gland not enlarged. Urine abundant. He had strange suffocative attacks every night.

It is not difficult to recognize here symptoms usually belonging to three distinct nervous affections, viz.:—hysteria, neurasthenia, and Graves's disease.

The association of hysteria and neurasthenia is common enough, but it is rare to have exophthalmic gôitre added to them; only one such case would appear to have been published. It is not impossible that complex nervous affections similar to the above are not as uncommon as the number of recorded cases would lead one to suppose.—*The Medical Chronicle*, *February*, 1891.

---

### CAMPHORIC ACID AND TELLURATE OF SODA IN NIGHT SWEATS. TWO NEW ANIDROTIC MEDICAMENTS.

Camphoric acid, which was discovered in 1675 by Lémery, has very recently been made the subject of clinical studies by Leu, in *Centralblatt für Klin. Med.*, and by Dreesmann, in an inaugural thesis. It is obtained by heating camphor with ten times its weight of nitric acid ; it presents itself in the form of little scales or colorless needles, which are transparent and bitter to the taste, melting at 70° C., and dissolving freely in alcohol, ether and essential oils, somewhat sparingly in boiling water, and being scarcely at all soluble in cold water.

Leu has remarked that, when camphoric acid is administered to tuberculous subjects who are subject to night sweats, in the dose of thirty grains, it produces a marked anidrotic effect several hours after its absorption ; sometimes the suppression of the sweats does not come on till the next day, and the effect of one dose may last several days. The same good results may be obtained from the use of alcoholic lotions of camphoric acid, the lotions being freely applied to parts where the sweating is localized. Leu affirms that no ill effects have ever followed the internal or external use of this remedy. A soporific action sometimes follows the ingestion of a thirty-grain dose.

Camphoric acid was tried in thirteen bad cases of night sweats ; in sixty per cent. of these cases the sweats were suppressed, in twenty-two per cent. they were diminished, and in eighteen per cent. only the remedy failed. Dreesmann has found this anidrotic effect of camphoric acid only in tuberculous cases ; if the sweats are due to any other cause, the remedy is inefficacious. His experiments have shown that the sweating, which is provoked by pilocarpine, is not influenced by camphoric acid ; hence he concludes that this medicament does not act by the intermediation of the central nervous system, but by destroying the soluble products of the tubercle bacillus, which are the direct cause of the profuse sweats symptomatic of the stage of pulmonary ulceration.

Combemale, in the *Bulletin Général de Thérapeutique*, January 15, 1891, has published the results of a series of clinical trials of camphoric acid, and concludes :

(1) That camphoric acid has a certain action on the night sweats of the tuberculous ; it very often arrests them, frequently diminishes them, and is very rarely without effect.

(2) These effects follow doses of thirty grains, once a day, at 7 o'clock P. M.

(3) No disagreeable or harmful after-effect has followed the usage of camphoric acid.

Combemale agrees with Leu as to the necessity of limiting the therapeutic application of the camphoric acid to the night sweats of the tuberculous, and affirms that in his experience it has a very uncertain action in advanced cases of pulmonary phthisis with large cavities. It is in recent cases where the purulent surfaces are small, where the tubercles are still crude, or are just beginning to soften, that it does the most good, and here the remedy is almost infallible in its action.

Another new anidrotic has been announced—tellurate of soda. It is an oxygenated salt of tellurium, and is obtained by heating a mixture of tellurium or its binoxide, with soda or nitrate of soda. It is the normal tellurate ($Na_2TeO_4+5H_2O$) which is employed. This salt is soluble in water and in alcohol, and presents itself after evaporation in the form of a gummy mass or whitish amorphous powder.

Neusser, in *Wiener Klin. Wochenschrift*, 1890, was the first to recommend the employment of tellurate of soda in night sweats. He gave pills of one-third of a grain each, and found one of these pills *per diem* sufficient to restrain the most obstinate night sweats of the phthisical. The ingestion of the remedy is followed by a garlic-smell of the breath, but no injurious effects have been noticed.

Combemale (*loc. cit.*) has made trials of tellurate of soda in phthisical cases ; he has obtained favorable results similar to those recorded by Neusser in nocturnal diaphoresis. He gave small doses, amounting to from half a grain to a grain daily, dissolved in a little julep. He has found that the remedy succeeds in the advanced stages of tuberculosis better than camphoric acid. The tellurate of soda acts equally well in restraining the the night sweats of rheumatism, typhoid fever and other prostrating affections. Both remedies are supposed to produce an antiseptic action, destructive of the soluble products of the microbes !—*The Boston Medical and Surgical Journal, February* 19, 1891.

